# Prediction of hyperkalemia in ESRD patients by identification of multiple leads and multiple features on ECG

**DOI:** 10.1080/0886022X.2023.2212800

**Published:** 2023-05-18

**Authors:** Daojun Xu, Bin Zhou, Jiaqi Zhang, Chenyu Li, Chen Guan, Yuxuan Liu, Lin Li, Haina Li, Li Cui, Lingyu Xu, Hang Liu, Li Zhen, Yan Xu

**Affiliations:** a Department of Nephrology, The Affiliated Hospital of Qingdao University, Qingdao, P.R. China; b Medizinische Klinik und Poliklinik IV, Klinikum der Universität, LMU München, München, Germany; c School of Artificial Intelligence, Sun Yat-sen University, Guangzhou, P.R. China

**Keywords:** Electrocardiogram, noninvasive hyperkalemia prediction, end-stage renal disease, machine learning, logistic regression

## Abstract

**Background:**

Patients with end-stage renal disease (ESRD) especially those undergoing dialysis have a high prevalence of hyperkalemia, which must be detected and treated immediately. But the initial symptoms of hyperkalemia are insidious, and traditional laboratory serum potassium concentration testing takes time. Therefore, rapid and real-time measurement of serum potassium is urgently needed. In this study, different machine learning methods were used to make rapid predictions of different degrees of hyperkalemia by analyzing the ECG.

**Methods:**

A total of 1024 datasets of ECG and serum potassium concentrations were analyzed from December 2020 to December 2021. The data were scaled into training and test sets. Different machine learning models (LR, SVM, CNN, XGB, Adaboost) were built for dichotomous prediction of hyperkalemia by analyzing 48 features of chest leads V2-V5. The performance of the models was also evaluated and compared using sensitivity, specificity, accuracy, accuracy, F1 score and AUC.

**Results:**

We constructed different machine models to predict hyperkalemia using LR and four other common machine-learning methods. The AUCs of the different models ranged from 0.740 (0.661, 0.810) to 0.931 (0.912,0.953) when different serum potassium concentrations were used as the diagnostic threshold for hyperkalemia, respectively. As the diagnostic threshold of hyperkalemia was raised, the sensitivity, specificity, accuracy and precision of the model decreased to various degrees. And AUC also performed less well than when predicting mild hyperkalemia.

**Conclusion:**

Noninvasive and rapid prediction of hyperkalemia can be achieved by analyzing specific waveforms on the ECG by machine learning methods. But overall, XGB had a higher AUC in mild hyperkalemia, but SVM performed better in predicting more severe hyperkalemia.

## 1. Introduction

Hyperkalemia is one of the most common electrolyte disturbances in clinical practice. According to the new standard, hyperkalemia is defined as a condition with serum potassium higher than 5.0 mmol/L. Factors that increase the risk of hyperkalemia include renal failure, diabetes mellitus, heart failure, adrenal disease and the use of angiotensin-converting enzyme inhibitors, angiotensin receptor blockers or potassium-sparing diuretics. Among them, the main risk factor for hyperkalemia is impaired renal function –either acute kidney injury (AKI) or advanced chronic kidney disease (CKD) [1–3].

Hyperkalemia increases the risk of death through multiple mechanisms. In addition to its obvious effect on cardiac excitability potentially leading to arrhythmias, hyperkalemia may also give rise to peripheral neuropathy and renal tubular acidosis. When using serum potassium as a continuous variable, the correlation between serum potassium levels and mortality was u-shaped, which implies that hyperkalemia is associated with a higher risk of death, whereas more severe CKD stages and levels of hyperkalemia may synergistically increase mortality [4–7]. When serum potassium concentration was >5.0 mmol/L, the more hyperkalemia occurs, the higher the mortality rate is [8].

Previous studies have shown that after hyperkalemia occurs in CKD patients, the interval between attacks will gradually shorten [9]. Increasing the frequency of monitoring can find more patients with hyperkalemia, while the lower frequency of serum potassium monitoring is prone to miss the diagnosis of patients with hyperkalemia, leading to adverse consequences [10]. Hyperkalemia may lead to changes in an electrocardiogram (ECG), such as T-wave tenting, PR interval prolongation, P-wave flattening, P-wave disappearance, QRS interval prolongation, etc. [11]. These patterns have been used by physicians qualitatively to infer probable hyperkalemia conditions, however, a computer-aided method that allows a more accurate diagnosis of hyperkalemia is seriously needed. With the development of science and technology, the machine learning method has more and more extensive applications in various disciplines in the field of medicine. Machine learning is emerging in areas such as the classification of images, ancillary diagnosis and treatment, and prediction of disease [12]. Therefore, we use different machine learning approaches to build practical prediction models to achieve non-hemorrhagic immediate prediction of serum potassium concentration by ECG, thus providing early warning of death due to sudden adverse consequences of hyperkalemia.

## 2. Methods

### 2.1. Inclusion and exclusion criteria

This study was conducted on patients who received standard hemodialysis regularly in the Affiliated Hospital of Qingdao University. This study was approved by the Institutional Ethics Committee (QYFY WZLL 27298) and the patient’s informed consent was obtained. All patients in this study received maintenance hemodialysis for more than three months, and four hours of hemodialysis three times a week. Of these, patients with vascular embolism disease, new cerebrovascular accident, heart failure, arrhythmia and acute coronary syndrome, acute cardiovascular events, previous cardiac surgery, pacemaker implantation, incomplete clinical data, and use of drugs that affect ECG conductions were excluded.

### 2.2. Serum potassium measurement

At the centre of this study, serum potassium concentration was regularly monitored monthly during dialysis and ECG was collected. Given the patients’ dialysis schedule, hyperkalemia was more likely to precede the first dialysis session of each week, so hospitals tended to schedule serum collections at the first hemodialysis session of each week. Blood was drawn at two**-**time points during each dialysis session: (1) before dialysis and (2) after dialysis, dialysate flow was stopped and the blood flow rate was reduced to 100 mL/min for at least 15 s. Immediately after blood collection, blood samples were sent to the laboratory for serum potassium analysis. Standardized procedures are performed during blood collection to prevent hemolysis from affecting serum potassium concentration.

### 2.3. ECG measurement

In all patients, 12-lead ECG data were obtained using standard electrode placement. ECG measurements were also performed at two-time points, one pre- and one post-dialysis. The interval between ECG measurements and blood sampling in the same patient should not exceed 5 min in order to maintain a good correlation between ECG and serum potassium concentrations.

### 2.4. Signal processing

ECG data collected by Nihon Kohden ECG-2550 were used as raw data. The original data is stored in PDF format. Refer to MIT-BIH arrhythmia database to save the data as a header file and data file, where the data file was stored in Format212 format, for the next step of data processing. After the initial treatment of the 12-lead data, we use the MATLAB R2018b tool to analyze and process the data.

**Figure 1.**
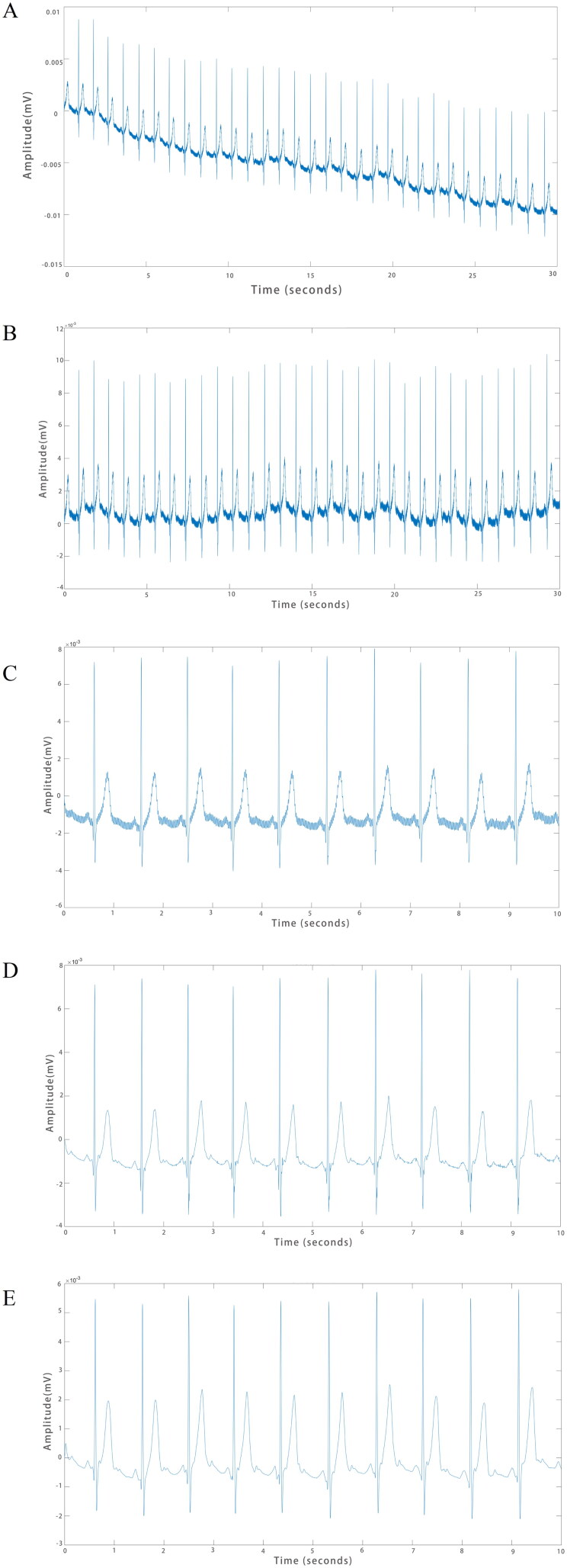
A preprocessing is required for the original ECG to become analyzable. (A) The original ECG. (B) ECGs after correction for mild baseline drift by polynomial fitting. (C) ECG after the second baseline correction. (D) ECG after wavelet transform. (E) ECG after bandpass filters.

The original ECG (Figure 1(A)) signal generally has baseline drift, power frequency interference and other noise, which needs data preprocessing before conducting data analysis. The mild baseline drift was corrected by polynomial fitting to the input ECG signals, and the corrected ECG data (Figure 1(B)) were obtained by subtracting the fitted function from the metadata. The length of the original ECG data was 30 s, and by liner fitting algorithm, the smoothest 10 s data were selected, and polynomial fitting was performed a second time, again correcting for baseline drift. (Figure 1(C)) Finally, the ECG signal was denoised through wavelet transform (Figure 1(D)) and band-pass filters (Figure 1(E)) to obtain ECGs that could be analyzed.

### 2.5. Feature extraction

For pretreatment ECGs, characteristic values on the ECG were extracted and calculated using MATLAB and Python software. After preceding repeated trials, the following three types of 12 ECG characteristics were selected among the initial multitude of characteristics: (1) characteristics of slope class: T right slope, T left slope, S-T band slope; (2) characteristics of amplitude class: T wave amplitude, R wave amplitude, S wave amplitude; and (3) characteristics related to area class: T wave area, R wave area, S wave area, T wave area per second, R wave area per second, S wave area per second. The above 12 ECG characteristics were extracted from V2-V5 on each of these four leads for a total of 48 feature values.

### 2.6. Model building

**Figure 2.**
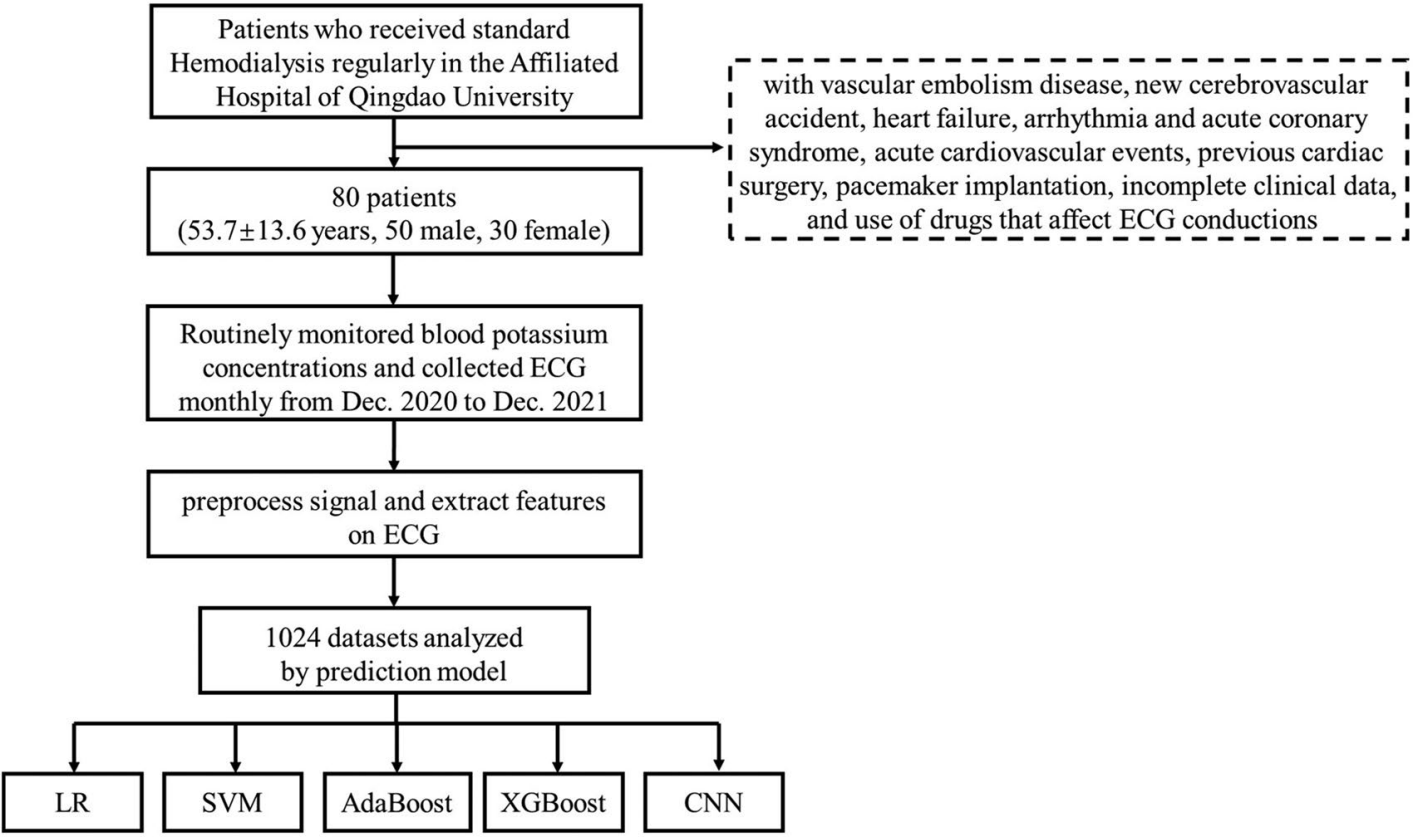
The flowchart of this study.

The 80 dialysis patients received ECG and blood tests every other month, from which we selected 1024 sets of matched data of serum potassium and ECG. To prevent data speculum bias, these 1024 sets of data were randomly divided into a training set and a test set in an 8:2 ratio before model building. In the training set, 5-fold cross-validation method was used to learn all the data sufficiently to finally determine the optimal hyperparameters of the individual models. For machine learning, four models of convolutional neural network (CNN), support vector machine (SVM), eXtreme Gradient Boosting (XGB) and Adaboost were compared. Logistic regression (LR) as a traditional clinical prediction model was also incorporated for comparison. Logistic regression is a generalized linear regression analysis model, which belongs to supervised learning in machine learning. It is effective and widely used in classification, especially in the study of secondary classification [13]. SVM is also a powerful supervised learning method, which is widely used in classification and regression, and is more robust than LR [14]. It has many unique advantages in solving small samples, nonlinear and high-dimensional pattern recognition. Adaboost is an integrated learning technique that trains multiple weak classifiers for the same training set and weights each classifier according to their errors, resulting in a strong classifier [15]. XGB is also a powerful supervised learning method which is a powerful gradient-boosted tree algorithm commonly used for regression, binary classification and multiclass classification problems [16]. CNN is a kind of feedforward neural network with convolution computation and depth structure, which was originally used for image recognition and classification [17]. Currently, it is widely used in the medical field for classifying images in imaging and pathology [18–20], voice recognition [21,22], etc. The structure of the CNN is shown in Figure S1, and the hyperparameters of the remaining 4 models are shown in Table S1. These models all output dichotomous outcomes, hyperkalemia and non-hyperkalemia. To evaluate the diagnostic ability of the model for different degrees of hyperkalemia, multiple prediction models were developed and compared by using serum potassium concentrations of 5.0, 5.5, 6.0, 6.5 mmol/L as the diagnostic threshold (Figure 2).

### 2.7. Evaluation indicators and statistical analysis

In order to evaluate the performance of different models, the main evaluation index is the area under the ROC curve (AUC). At the same time, the sensitivity, specificity, accuracy, accuracy and F1 score of these five models were calculated. Differences between AUCs were compared using the Delong test. Two-sided P values less than 0.05 were considered statistically significant. All statistical analyses were performed by Python (version 3.7, Python Software Foundation, Wilmington, DE).

## 3. Results

Based on the above criteria, we finally selected 80 patients for data collection from December 2020 to December 2021. We collected information on these 80 hemodialysis patients when data collection was initially initiated and the results are shown in Table 1. These 80 patients included 50 males and 30 females, and their mean age was 53.7. At the beginning of data collection, the average number of years of hemodialysis for these people was 3, and the average serum creatinine (Scr) of these patients was 992.3 μmol/L. Most of these individuals (86.3%) had hypertension.

**Table 1.** Characteristics of 80 patients.

Characteristic	All patients (*N* = 80)
Gender, *n* (%)	
Male	50 (62.5)
Female	30 (37.5)
Age (years), mean (SD)	53.7 (13.6)
Height (cm), mean (SD)	168.9 (8.2)
Weight (kg), mean (SD)	66.9 (12.4)
BMI (kg/m^2^), mean (SD)	23.4 (3.3)
Average time of dialysis (year), mean (SD)	3.0 (2.7)
Scr (μmol/L), mean (SD)	992.3 (273)
Bun (mmol/L), mean (SD)	27.3 (7.3)
kt/v, mean (SD)	1.5 (0.1)
HCO_3_^-^ (mmol/L), mean (SD)	20.5 (2.8)
Comorbidities, *n* (%)	
Diabetes mellitus	11 (13.8)
Coronary artery disease	12 (15)
Hypertension	69 (86.3)
Cerebral infarction	4 (5)

**Figure 3.**
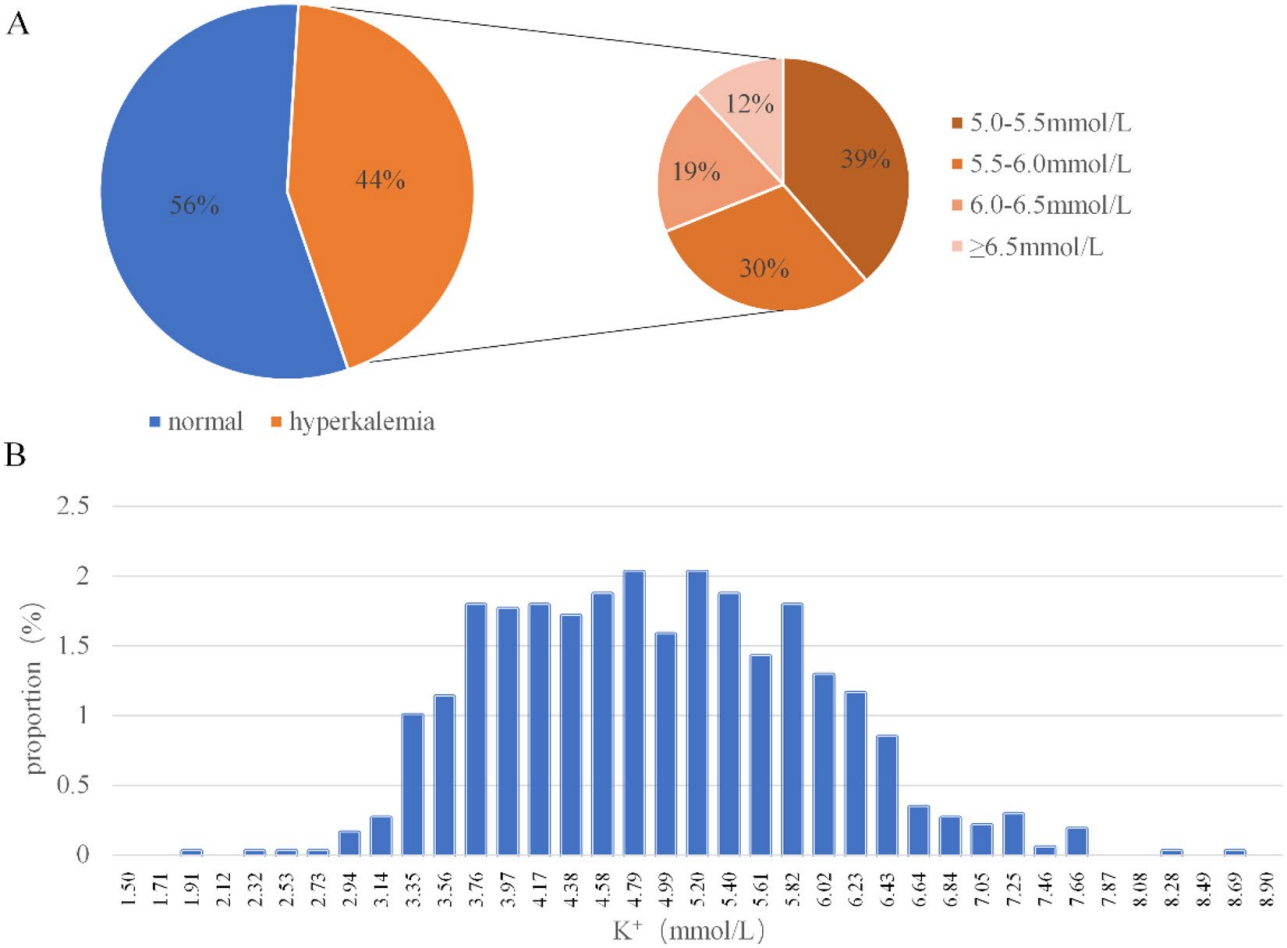
Distribution of 1024 blood potassium concentration results.

A total of 1024 sets of serum potassium concentration and ECG data sets were included in this study. The mean potassium concentration of these 1024 datasets was 4.83 ± 1.01 mmol/L. The average values of different characteristics on each lead are shown in Table S2. Among them, 576 had serum potassium concentration less than 5.0 mmol/L, 173 had serum potassium concentration greater than or equal to 5 mmol/L and less than 5.5 mmol/L, 136 had serum potassium concentration greater than or equal to 5.5 mmol/L and less than 6.0 mmol/L, 85 had serum potassium concentration greater than or equal to 6.0 mmol/L and less than 6.5 mmol/L, and 54 had serum potassium concentration greater than or equal to 6.5 mmol/L. The concentration distribution of potassium ions is relatively concentrated between 3.5 mmol/L and 6.0 mmol/L (Figure 3). The prevalence of hyperkalemia was 43.8% when 5.0 mmol/L was used as the threshold for blood potassium concentration. When 5.5 mmol/L was used as the threshold for hyperkalemia, the prevalence was 26.9%. The prevalence of hyperkalemia with a blood potassium concentration above 6.0 mmol/L was 13.6%, and the prevalence of severe hyperkalemia with a blood potassium concentration above 6.5 mmol/L was 5.3%.

The sensitivity, specificity, accuracy, precision, and F1 score of the different models at different serum potassium concentration thresholds were varied (Table 2). For the same machine learning method, the same evaluation indicators decreased as the threshold of serum potassium concentration increased. For instance, when 5.0 was used as the threshold concentration for hyperkalemia, the models performed more consistently and excellently. Taking CNN as an example, when using 5.0 mmol/L as the concentration threshold for hyperkalemia, the accuracy, precision, sensitivity, specificity and F1 score of the model were 0.815, 0.769, 0.851, 0.784 and 0.808. The F1 scores of CNN (0.808), SVM (0.812), XGB (0.861), and AdaBoost (0.844) performed better than LR (0.781) when a blood potassium concentration of 5.0 mmol/L was used as the diagnostic threshold for hyperkalemia. Actually, when using 5.0 or 5.5 as diagnostic thresholds, the AUC and F1-score of LR were inferior to the other four machine learning methods.

**Table 2.** Performance of different models at different serum potassium concentration thresholds.

	Threshold of hyperkalemia (mmol/L)	Accuracy	Precision	Sensitivity	Specificity	F1-score	AUC
CNN	5	0.815	0.769	0.851	0.784	0.808	0.900
	5.5	0.717	0.495	0.930	0.635	0.646	0.824
	6	0.834	0.395	0.577	0.872	0.469	0.829
	6.5	0.741	0.155	0.692	0.745	0.254	0.754
SVM	5	0.820	0.777	0.851	0.793	0.812	0.892
	5.5	0.829	0.696	0.684	0.885	0.690	0.861
	6	0.810	0.362	0.654	0.832	0.466	0.784
	6.5	0.883	0.261	0.462	0.911	0.333	0.781
XGB	5	0.863	0.806	0.926	0.811	0.861	0.931
	5.5	0.824	0.672	0.719	0.865	0.695	0.872
	6	0.883	0.542	0.500	0.939	0.520	0.806
	6.5	0.922	0.364	0.308	0.964	0.333	0.751
AdaBoost	5	0.863	0.884	0.809	0.910	0.844	0.923
	5.5	0.771	0.563	0.789	0.764	0.657	0.831
	6	0.878	0.526	0.385	0.950	0.444	0.809
	6.5	0.922	0.364	0.308	0.964	0.333	0.740
LR	5	0.795	0.765	0.798	0.793	0.781	0.858
	5.5	0.815	0.702	0.579	0.905	0.635	0.824
	6	0.873	0.500	0.538	0.922	0.519	0.823
	6.5	0.898	0.300	0.462	0.927	0.364	0.744

**Figure 4.**
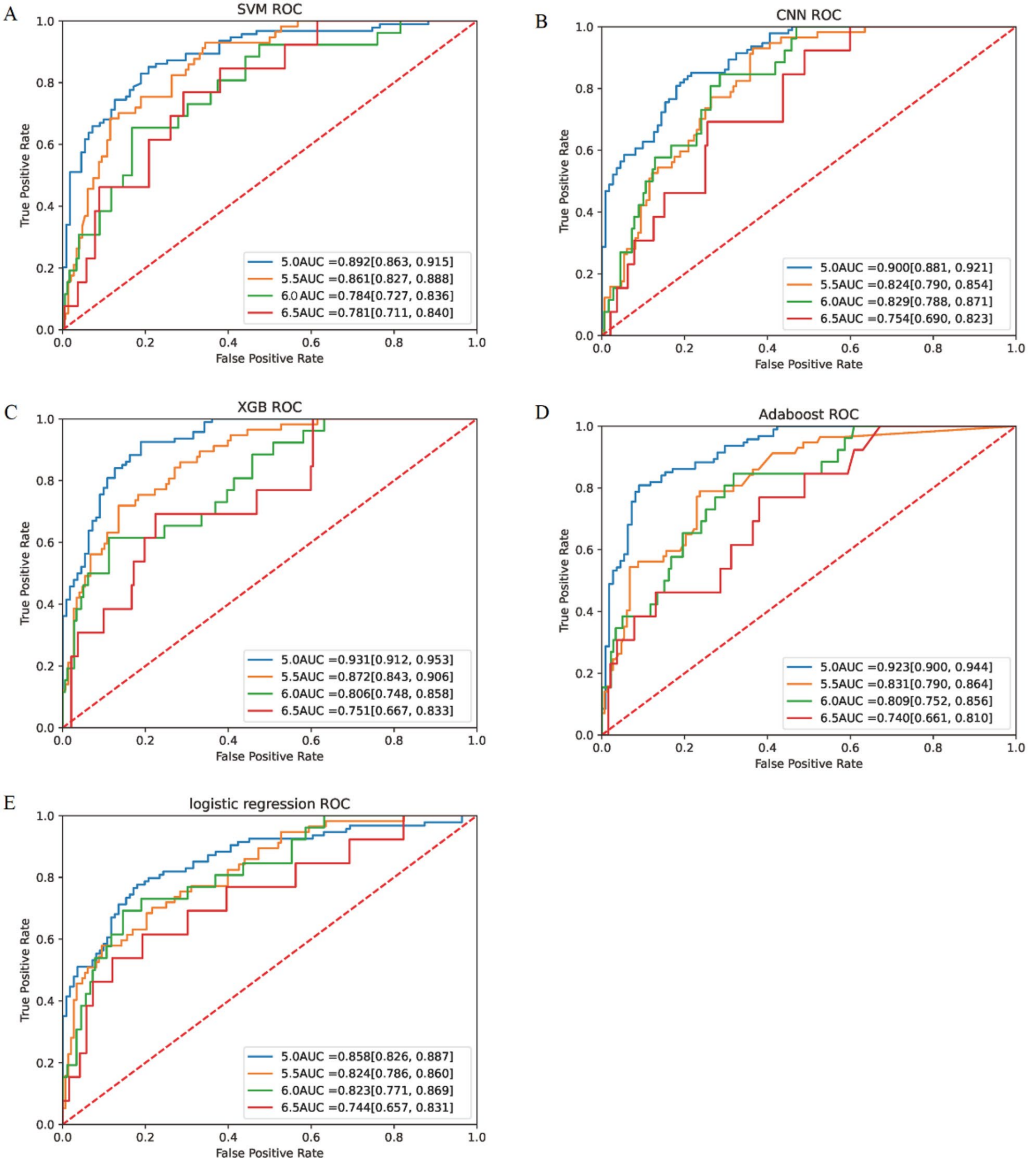
ROC of different machine learning models for different degrees of hyperkalemia.

**Figure 5.**
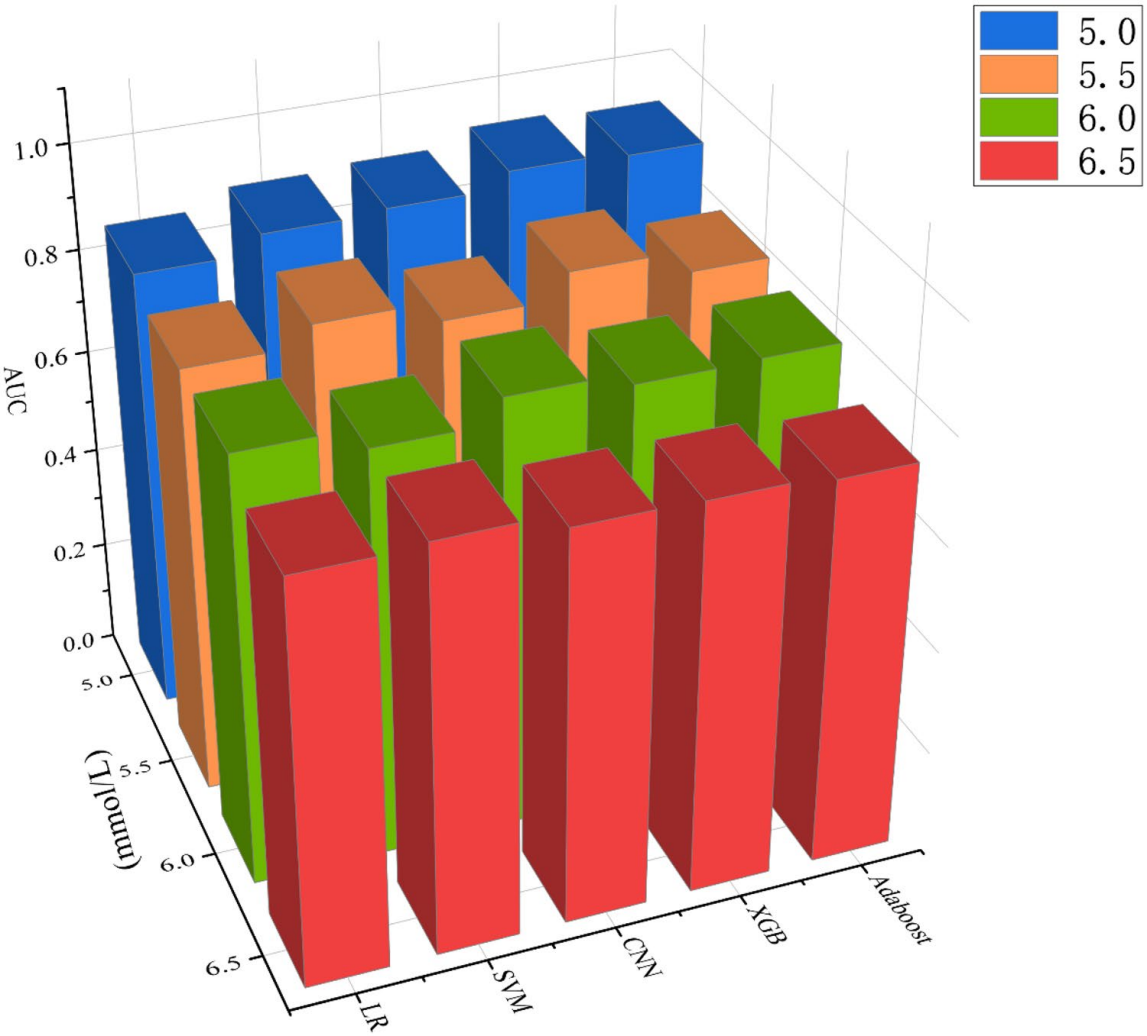
Comparison of AUC of machine learning models at different hyperkalemia concentration thresholds.

When serum potassium concentration higher than 5.0 mmol/L was used as the diagnostic criterion for hyperkalemia, the AUC was 0.892 (95%CI, 0.863–0.915) for SVM, 0.900 (95%CI, 0.881–0.921) for CNN, 0.931 (95%CI, 0.912–0.953) for XGB, and 0.923 (95%CI, 0.900–0.944) for Adaboost. However, the LR had a slightly lower AUC of 0.858 (95%CI, 0.826–0.887), which was significantly lower than other models (*p* < 0.05). When using the current more prevalent diagnostic criteria for hyperkalemia, that is, serum potassium concentration over 5.5 mmol/L as the standard, the AUC was 0.861 (95%CI, 0.827–0.888) for SVM, 0.824 (95%CI, 0.790–0.854) for CNN, 0.872 (95%CI, 0.843–0.906) for XGB, 0.831 (95%CI, 0.790–0.864) for Adaboost, and 0.824 (95%CI, 0.786–0.860) for LR. In order to compare the predictive ability of different learning models for different degrees of hyperkalemia, the above model was additionally trained with serum potassium concentrations of 6.0 mmol/L and 6.5 mmol/L as diagnostic thresholds. When serum potassium concentration was 6.0 mmol/L as the diagnostic criteria for severe hyperkalemia, the AUCs of SVM, CNN, XGB, Adaboost and LR were 0.784 (95%CI, 0.727–0.836), 0.829 (95%CI, 0.788–0.871), 0.806 (95%CI, 0.748–0.858), 0.809 (95%CI, 0.752–0.856), 0.823 (95%CI, 0.771–0.869), respectively. The diagnostic power of different models for extreme hyperkalemia varied considerably. When dichotomous prediction was performed with serum potassium concentration 6.5 mmol/L as the diagnostic threshold, the AUC of Adaboost was 0.740(95%CI, 0.661–0.810), and that of LR was 0.744 (95%CI, 0.657–0.831). The best performing of these models was SVM, whose AUC was the highest among the models with 6.5 as the threshold, reaching 0.781 (95%CI, 0.711–0.840**)**. (Figure 4(A–E)) For the same model, with the gradual aggravation of hyperkalemia, AUC gradually decreased (Figure 5).

## 4. Discussion

Hyperkalemia, an invisible killer in the clinic, has a high prevalence, especially in people with renal failure. However, the traditional method to detect serum levels of potassium by blood sampling often takes time to obtain the results, and cannot meet the requirement of multiple, real-time detection and management of serum potassium. How to quickly identify hyperkalemia at an early stage before admission and how to more conveniently carry out long-term monitoring of hyperkalemia in high-risk individuals with hyperkalemia are urgent issues. ECG, as a basic and comparable test for outpatients and inpatients, its waveform is affected by the change in potassium ion concentration in the body. Therefore, feature extraction from the ECG waveform and analysis using machine learning methods can achieve noninvasive and rapid prediction of hyperkalemia. The usual definition of hyperkalemia is a blood potassium concentration >5.5 mmol/L. The KDIGO [23] expert consensus on blood potassium management in renal disease recommends updating the definition of hyperkalemia to serum potassium >5.0 mmol/L. Guidelines indicate that emergency potassium lowering therapy is required when serum potassium concentrations >6 mmol/L. This study used these common classification methods to predict different degrees of hyperkalemia, and compared AUC, recall rate, specificity and other evaluation indicators between different methods. When looking at common hyperkalemia with serum potassium concentrations greater than 5.0 mmol/L, the five machine learning methods included in this study performed better for sensitivity, specificity, accuracy, precision, F1 score as well as AUC, and better for other degrees of hyperkalemia.

Basal narrow and high sharp T waves can be present on the ECG of patients with mild to moderate hyperkalemia, which is also the earliest and most common ECG change in this subset of hyperkalemia patients [11,24,25]. When the extracellular potassium ion concentration is higher, equivalent to when the cell membrane is in a hyperpolarized state. Inwardly rectifying potassium channels in atrial myocytes increases potassium permeability, which accelerates repolarization. So the T wave base narrowed and the waveform became tall, peaked, and tented [26–28]. In addition to T wave changes, patients with hyperkalemia may exhibit S-T segment elevation, PR interval prolongation, QRS duration prolongation, absent P waves, and sinusoidal waveforms on their ECG [29–31]. On the other hand, hyperkalemia can also cause some atypical ECG changes, such as T wave inversion, ST segment depression, etc. [3]. Therefore, this study has tried to include as comprehensively as possible the features of area class, slope class, interval class and amplitude class associated with T wave, P wave, QRS wave, ST segment, etc. We did not include all chest leads because we observed that some waveforms on leads V1 and V6 did not perform as well as the other four chest leads. The waveform of the electrocardiogram will be affected by subjects’ mental and psychological factors, abnormal breathing [32], body temperature [33,34], chest lead electrode pressure and lead position [35], etc., so these accidental factors will have an impact on the prediction results of the prediction model.

Zubaid et al. [36] collected 528 ECGs from ESRD patients presenting to the emergency department and found that the mean sensitivity and specificity for detecting hyperkalemia by the emergency physician were 0.19 and 0.97, respectively. In severe hyperkalemia with serum potassium concentrations above 6.5 mmol/L, the average sensitivity improved to 0.29, while the specificity decreased to 0.95. It can be seen that when clinicians empirically predict hyperkalemia by analyzing the ECG, the sensitivity is often inadequate, the diagnosis is prone to be missed and the likelihood of associated adverse events increases. Brian et al. [37] studied ECG in patients with blood potassium concentrations above 6.0 mmol/L and concluded that electrocardiograms were not sensitive to the diagnosis of hyperkalemia and only approached the minimum predictive ability at potassium levels of 7.2–9.4 mmol/L. Darren et al. [38] also showed that both T- and R-wave amplitudes on the ECG were less sensitive in detecting hyperkalemia in ESRD patients. Limei Zhou et al. [39] previously included 401 samples to construct a prediction model by multiple logistic regression, using the number of hemodialysis sessions, blood urea nitrogen (BUN), serum sodium, serum calcium, serum phosphorus and diabetes mellitus to identify hyperkalemia and predict serum potassium concentrations higher than 5.5 mmol/L. The AUC of this model was 0.82 (0.77, 0.88) in the training set and 0.81 (0.74, 0.88) in the validation set. Previously, studies have utilized different machine learning methods and different ECG leads and features to predict hyperkalemia. Dennis et al. [40] used the general linear mixed model to diagnose hyperkalemia by analyzing the 12-lead ECG, and finally established a model incorporating the T-wave width, descending T-wave slope and new QRS prolongation. The AUC in the validation set was 0.78. The maximum specificity and sensitivity of the model for serum potassium above 5.91 mmol/L were 84% and 63% respectively. The inclusion criterion for the study was a time interval of less than 4 h between the measurement of potassium concentration and the 12 lead ECG, but this time interval is too long was an inadequacy of the study because the interventions given during this period may have changed the ECG. Also defining the time gap of inclusion criteria as 4 h is the study of Friedman et al. [41] which previously used 1,576,581 ECGs from 449,380 patients seen at Mayo Clinic, Rochester, Minnesota, from 1994 to 2017, as a training set and 5.5 mmol/L as the diagnostic threshold to predict hyperkalemia by learning 2 (leads I and II) or 4 (leads I, II, V3, and V5) ECG leads using a deep convolutional neural network model. And validated using retrospective data from the Mayo Clinic in Minnesota, Florida, and Arizona. In this study, using only 2 ECG leads, the deep learning model detected hyperkalemia in renal disease patients with an AUC of 0.853 to 0.883. However, in our study, specific features on leads V2 to V5 were converted to digital format for subsequent model training, achieving a better AUC in the prediction of mild hyperkalemia. And, the criterion for the inclusion of data in this study was that the interval between ECG examination and blood sampling did not exceed 5 min in order to maintain a good correlation between ECG and blood potassium concentration.

In the present study, when using a serum potassium concentration of 5.0 mmol/L as the diagnostic threshold for hyperkalemia, the other 4 machine models had similar AUCs and were significantly better than LR. And the AUCs of all five machine learning methods were highest at that point, probably because the data distribution was more balanced when blood potassium concentration 5.0 was used as the threshold value, which was more conducive to the model for feature learning. As the diagnostic threshold increased, the AUCs of the models decreased to different degrees. This may be because the data for extreme hyperkalemia decreased as the diagnostic threshold increased, and the imbalance in the data resulted in lower model power [42]. This situation will improve to some extent in the future as more data are collected and the population involved increases. The AUC of XGB was highest in severe hyperkalemia with serum potassium concentrations above 5.0 and 5.5mmol/L, which suggested that XGB was preferable in the case of mild hyperkalemia. In previous studies by Zhang et al. [43] and Saraiva et al. [44], XGB also had better performance in the comparison of models. But in extreme hyperkalemia with serum potassium concentration higher than 6.5mmol/L, SVM had higher AUC, which might be related to the small sample size of extreme hyperkalemia. This study only made the comparison between different models, while the ensemble model developed by fuzing multiple machine learning algorithms was able to have a better calibration and discrimination ability [45].

This study excluded patients with diseases that may cause ECG changes. Different results and conclusions may be obtained in these populations, and the universality of the model was insufficient. In addition, this study lacks relevant biochemical parameters, age, sex and other information, and only uses ECG to train the model, which may lead to other factors of hyperkalemia not being included in the model, so the ability of the model was not further improved. Due to conditional limitations, this study included fewer patients. On the other hand, the present study, being a prospective study, strictly limited the time interval between ECG and blood sampling to 5 min, although it could be ensured that the time interval between ECG and blood sampling had no other contributing factors to the ECG. However, this also resulted in a smaller sample size for acquisition and a smaller sample size for severe hyperkalemia, leading to inadequate model learning and uneven sample distribution, all of which may explain the poor performance of these five models in predicting severe hyperkalemia. Another weakness is that this study was a single-center study and lacked the results of external data validation.

## 5. Conclusion

In conclusion, although the performance of the different models is related to the severity of hyperkalemia, and these models all performed better in predicting mild hyperkalemia with concentrations greater than 5 mmol/L, at this time XGB, AdaBoost, SVM and CNN performed significantly better than LR. XGB had a higher AUC in mild hyperkalemia, but SVM performed better in predicting more severe hyperkalemia.

## Supplementary Material

Supplemental Material
