# Supplementary material for: Prediction of hyperkalemia in ESRD patients by identification of multiple leads and multiple features on ECG
Source: Ren Fail. 2023 May 18;45(1):2212800. doi: 10.1080/0886022X.2023.2212800 (PMC10197982; doi:10.1080/0886022X.2023.2212800)
Supplement: Supplemental Material [file IRNF_A_2212800_SM9119.pdf]

# Supplementary material

**Figure S1. Structure of CNN**

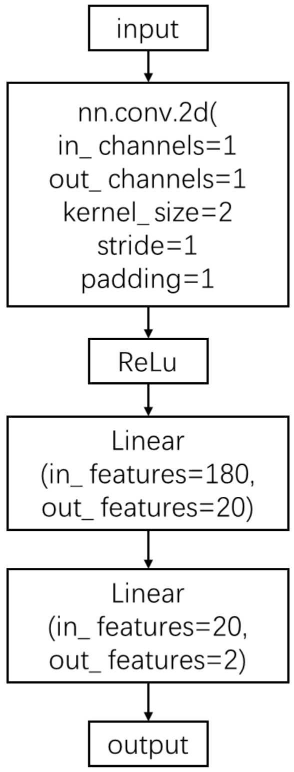

**Table S1. Superparameters of AUC under different serum potassium concentration thresholds.**

| | | Hyperparameters optimized | Best hyperparameter values (5.0mmol/L) | Best hyperparameter values (5.5 mmol/L) | Best hyperparameter values (6.0 mmol/L) | Best hyperparameter values (6.5 mmol/L) |
|---|---|---|---|---|---|---|
| SVM | Kernel | "poly" | | | | |
| | cache_size | 200 | | | | |
| | max_iter | 500000 | | | | |
| | decision_function_shape | "ovr" | | | | |
| | Gamma | "auto" | | | | |
| | C | 1,2,5 | 2 | 1 | 5 | 1 |
| | degree | 1,2,3,4 | 1 | 1 | 1 | 1 |
| | coefo | 0.0,0.1,0.5,1 | 0.0 | 0.1 | 0.0 | 0.0 |
| | tol | 0.001,0.01,0.1,1 | 1 | 0.1 | 0.001 | 0.001 |
| XGB | min_child_weight | 1 | | | | |
| | gamma | 0 | | | | |
| | learning_rate | 0.0001,0.001,0.01,0.1,0.5,1 | 0.1 | 0.1 | 0.01 | 0.01 |
| | n_estimators | 16,32,64,128,256,512,1024,2048 | 64 | 512 | 512 | 512 |
| | max_depth | 3,4,5,6,7,8,9,10 | 5 | 3 | 3 | 3 |
| AdaBoost | base_estimator | DecisonTreeClassifier | | | | |
| | algorithm | "SAMME" | | | | |
| | max_depth | 4,5,6,7,8 | 8 | 4 | 8 | 6 |
| | min_samples_split | 2,3,4,5 | 2 | 5 | 2 | 2 |
| | min_samples_leaf | 2,3,4 | 3 | 2 | 2 | 3 |
| | n_estimators | 20,25,30,35,40 | 35 | 40 | 35 | 35 |
| | learning_rate | 0.05,0.1,0.2 | 0.2 | 0.05 | 0.2 | 0.2 |
| Logistic Regression | penalty | "l2" | | | | |
| | C | 0.1,0.5,1,2 | 0.1 | 1 | 0.5 | 1 |
| | tol | 1e-6,1e-5,0.0001,0.001 | 1e-6 | 1e-6 | 1e-6 | 1e-6 |

Information Classification: General

**Table S2. Baseline table of blood potassium concentration and features on ECG for 1024 datasets.**

| characteristic | Mean ± SD |
|---|---|
| potassium concentration | 4.83±1.01 |
| T left slope_V2 | -0.01±0.01 |
| T left slope_V3 | -0.02±0.01 |
| T left slope_V4 | -0.01±0.01 |
| T left slope_V5 | -0.01±0.01 |
| T right slope_V2 | 0.53±0.34 |
| T right slope_V3 | 0.52±0.34 |
| T right slope_V4 | 0.40±0.32 |
| T right slope_V5 | 0.29±0.28 |
| S-T band slope_V2 | 0.01±0.01 |
| S-T band slope_V3 | 0.01±0.01 |
| S-T band slope_V4 | 0.01±0.01 |
| S-T band slope_V5 | 0.01±0.01 |
| R wave amplitude_V2(mV) | 0.44±0.34 |
| R wave amplitude_V3(mV) | 0.82±0.53 |
| R wave amplitude_V4(mV) | 1.12±0.56 |
| R wave amplitude_V5(mV) | 1.20±0.51 |
| S wave amplitude_V2(mV) | 0.05±0.03 |
| S wave amplitude_V3(mV) | 0.05±0.03 |
| S wave amplitude_V4(mV) | 0.06±0.03 |
| S wave amplitude_V5(mV) | 0.06±0.03 |
| T wave amplitude_V2(mV) | -1.17±0.67 |
| T wave amplitude_V3(mV) | -0.84±0.60 |
| T wave amplitude_V4(mV) | -0.54±0.46 |
| T wave amplitude_V5(mV) | -0.31±0.33 |
| R wave area_V2($mm^2$) | 411.24±48.01 |
| R wave area_V3($mm^2$) | 413.67±48.35 |
| R wave area_V4($mm^2$) | 415.22±50.20 |
| R wave area_V5($mm^2$) | 415.64±52.55 |
| S wave area_V2($mm^2$) | 123.72±16.73 |
| S wave area_V3($mm^2$) | 123.53±14.29 |
| S wave area_V4($mm^2$) | 122.23±15.08 |
| S wave area_V5($mm^2$) | 122.38±16.89 |
| T wave area_V2($mm^2$) | 175.18±35.30 |
| T wave area_V3($mm^2$) | 180.21±34.10 |
| T wave area_V4($mm^2$) | 183.86±33.17 |
| T wave area_V5($mm^2$) | 188.86±35.41 |
| R wave area per second_V2($mm^2/s$) | 162.27±90.76 |
| R wave area per second_V3($mm^2/s$) | 156.98±92.87 |
| R wave area per second_V4($mm^2/s$) | 126.54±82.72 |

Information Classification: General

| | |
|---|---|
| R wave area per second_V5($mm^2/s$) | 99.08±65.00 |
| S wave area per second_V2($mm^2/s$) | 130.86±100.97 |
| S wave area per second_V3($mm^2/s$) | 243.29±156.25 |
| S wave area per second_V4($mm^2/s$) | 333.98±165.89 |
| S wave area per second_V5($mm^2/s$) | 357.42±151.08 |
| T wave area per second_V2($mm^2/s$) | 342.02±205.83 |
| T wave area per second_V3($mm^2/s$) | 249.33±180.31 |
| T wave area per second_V4($mm^2/s$) | 161.00±139.18 |
| T wave area per second_V5($mm^2/s$) | 93.11±97.88 |

Information Classification: General
